# The Profession of British Nurses: Plain Words on Registration.—III

**Published:** 1917-04-14

**Authors:** 


### THE MATRONS' AND SISTERS' DEPARTMENT.

### THE PROFESSION OF BRITISH NURSES.

### Plain Words on Registration.—III.*

THE MIDWIVES ACT.

The Midwives Act corresponds with the Medical Act in that it was passed as a result of clamour, and solely for the protection of the public. For full sixty years after Charles Dickens had introduced Mrs. Gamp to the world as the personification of incompetence, ignorance, and intemperance, that lady plied her trade unhindered. "Don't ask me whether I won't take more, or whether I will, but leave the bottle on the chimney-piece, and let me put my lips to it when I am so dispoged." But the Acts differ in this important respect, that whereas the Medical Act allows quacks and empirics to practise in certain conditions, the law is absolute and uncompromising concerning the midwife. "No woman shall habitually and for gain attend women in childbirth otherwise than under the direction of a qualified medical practitioner unless she be certified under this Act."

Now the value to a profession of a State Register is in precise proportion to the extent to which it confers a monopoly of employment on those whose names are on the roll. If the employment of unregistered persons proceeds as before, the economic value of the Register to qualified persons is nil. On this account the importance to midwives of State Registration cannot be exaggerated. In passing the Act Parliament was in no respect prompted by this consideration, the motive of the Legislature being solely to ensure the honest and deserving poor from being the victims of the Gamp species. To allow a loophole would have been fatal to the success of the Act, and the full result is that large numbers of unqualified and incompetent midwives have migrated to Ireland to carry on their nefarious trade, this being the only part of the kingdom which has deliberately remained aloof. The creation of a Roll of Midwives for Great Britain has put uncertified women out of business. This fact demands the careful consideration of nurses seeking after Registration. If it could be demonstrated that it is possible to pass an Act for general nurses corresponding with the Midwives Act the ardent advocates of Registration would be like the Pied Piper of Hamelin. Every mother's daughter of the profession would flock to their banner, and the whole of the British constabulary would become their declared allies. It so happens, however, that midwifery differs radically from every other class of nursing. It consists of duty which the midwife must be competent to perform without the assistance of a doctor. She must be highly qualified for this important work, and be fully aware in what circumstances it is desirable or necessary to send for a doctor. The most highly trained and efficient general nurse occupies a fundamentally different position from this. The first article of her professional creed enunciates that her efficiency depends on strict observance of the doctor's orders. When the examiner asks St. John Ambulance candidates what they would do in certain circumstances the astute student replies, "Send for a doctor, Sir!" Similarly with the trained nurse. Save in cases of emergency, no matter how skilled or highly trained a nurse may be, to act independently is heresy, and the public would resent such action at least as strongly as the medical profession. The *raison d'être* of the midwife, on the other hand, is that she takes the doctor's place in normal cases of childbirth, and for this function the State properly insists that she is qualified, and insists likewise that none save qualified persons are permitted to receive payment for their services.

The close association of the nursing profession with doctors and with midwives has given the Registration movement a strong impetus, and has led its supporters to the erroneous conclusion that State Registration and monopoly of employment are of necessity in close relationship. The more carefully the inquiry is pursued the more clearly will it appear that the State will not, and, moreover, that it cannot, prevent untrained nurses from acting as they do now under medical supervision. This we propose categorically to demonstrate. In the meantime our readers will find it instructive to note the attitude of the Legislature toward the maternity or monthly nurse. The Midwives Act makes it quite clear that untrained or unqualified persons may perform the duties of maternity nurse without hindrance so long as they act under medical supervision. Maternity nurses learn their duties and acquire their certificates, not in order to conform with the law, but because they are compelled to do so by the conditions of the market. The higher their qualifications the better their fees and the less precarious their employment. For similar reasons general nurses undergo long periods of training in first-class hospitals.

Our arguments on this important and interesting subject do not constitute either a policy or a doctrine. We are engaged in an effort to distinguish fact from fiction, to enable the rank and file of the profession to see with clearer vision, and to save them from the danger of being lured into a fool's paradise.

* For previous articles see March 31, p. 521, and April 7, p. 15.

